# Clinical outcomes of COVID-19 in hemodialysis patients

**DOI:** 10.3389/fmed.2023.1281594

**Published:** 2023-11-13

**Authors:** Lina Adwan, Tala Al-Sadi, Shorouq Shawakha, Ni’meh A. Al-Shami

**Affiliations:** Department of Pharmacy, College of Pharmacy Nursing and Health Professions, Birzeit University, Birzeit, Palestine

**Keywords:** chronic kidney disease, COVID-19, hemodialysis, mortality, risk factor

## Abstract

**Background:**

The coronavirus disease 2019 (COVID-19) is known for its effects on the respiratory system. Three years after the pandemic morbid and mortal consequences, growing evidence is showing that the disease also has adverse outcomes and complications on additional organs including the kidneys. This study aims at investigating the effects of COVID-19 on hemodialysis patients receiving services at Palestine Medical Complex (PMC) kidney dialysis department, and to identify mortality related risk factors.

**Methods:**

In April 2022, data was collected using the electronic medical records system for the dialysis department at PMC. The study included all PMC hemodialysis patients that were infected with COVID-19 between January 2020–April 2022. The collected data included patient demographics, clinical features, laboratory tests, dialysis frequency and the disease outcome.

**Results:**

The results showed that the patients’ outcomes and dialysis frequency were impacted by their blood urea nitrogen (BUN), serum creatinine (SCr) and calcium levels. About one third of the study population died after being infected with COVID-19. The frequency of dialysis was also affected by the presence of comorbidities like hypertension, diabetes mellitus (DM) and myocardial infarction (MI).

**Conclusion:**

This study found that there was a high mortality rate within the hemodialysis patients infected with COVID-19. Having comorbidities affected the frequency of dialysis following COVID-19 infection. Dialysis patients should be protected from infections such as COVID-19 and their comorbidities should be monitored and kept under control as much as possible.

## 1. Introduction

Coronavirus disease 2019 (COVID-19) is one of the epidemic infections caused by the severe acute respiratory syndrome coronavirus 2 (SARS-CoV-2), it was declared as a global pandemic in 2020 which was followed by its devastating health, social, and economic consequences. Since then, the health impact of COVID-19 has been examined on the general population as well as the more vulnerable patients. In 2021, COVID-19 was the leading cause of death in the West Bank accounting for 25.2% of deaths, followed by cardiovascular diseases and cancer, which were responsible for 23.7 and 12.2% of deaths respectively, the fatality rate of COVID-19 in 2021 in Palestine was 1.25% (1).

COVID-19 is one of the beta-coronavirus group, consisting of a single-stranded positive-sense RNA genome and 4 structural proteins (S, E, M, and N). N is for the nucleocapsid protein that covers the genome in addition to another envelope that is associated with membrane protein (M), (S) for Spike protein and E the envelope protein (2). SARS-CoV-2 mainly enters the human body via the respiratory pathway either directly by respiratory droplets from sneezing and coughing or indirectly by contaminated surfaces. It is very contagious, droplets typically reach about two meters and can stay suspended in the air for three hours. Also, it can spread indirectly via touching contaminated surfaces then to the mucous membranes in the nose, eyes or mouth (3).

Since COVID-19 mainly affects the respiratory tract, its common symptoms include fever, non-productive cough and shortness of breath (SOB), in addition to fatigue, headache, dizziness, nausea and vomiting (N/V), and pain in joints and muscles. The average of COVID-19 incubation period is about 5 days which can vary depending on the patient health status and the virus variant, it can be severe and can have longer duration for patients with other underlying diseases such as respiratory failure, congestive heart failure (CHF), myocardial infarction (MI), arrhythmias, acute or chronic kidney disease (CKD) or liver failure (4, 5).

COVID-19 can also affect other organs. The kidney is one of the affected organs since the virus can bind with angiotensin converting enzyme (ACE) receptors in the lungs and kidneys (6). CKD is classified into five stages according to kidney disease improving global outcomes (KDIGO) classification that is based on the estimated glomerular filtration rate (eGFR), which estimates how much blood passes through the glomeruli per minute (7). GFR is affected by age, gender, and patient serum creatinine (SCr) levels. After measuring the GFR, stage can be identified as: Stage 1 CKD, mild kidney damage (eGFR more than 90 milliliters per minute); Stage 2, mild damage (eGFR = 60 to 89 milliliters per minute); Stage 3a, moderate damage (eGFR = 45 to 59 milliliters per minute); Stage 3b, moderate damage (eGFR = 30 to 44 milliliters per minute); Stage 4, severe damage (eGFR = 15 to 29 milliliters per minute); and Stage 5, kidney failure (eGFR less than 15). COVID-19 has different intensity and complications which increase depending on the stage, especially in patients at the end stage of renal disease that require replacement therapy including dialysis (8).

There are two types of dialysis, hemodialysis and peritoneal dialysis, they both act as a replacement of kidney functions of filtering waste and excess fluid from the blood. Hemodialysis, the most common type used, uses a machine that takes blood from the body, filters it through a dialyzer and returns the cleaned blood to the body. Hemodialysis is performed for patients with end stage CKD having GFR of less than 15. The process takes approximately 4 h and commonly takes place at hospitals or at a dialysis center.

Patients with advanced age who are on dialysis already have comorbid conditions such as hypertension, diabetes mellitus (DM), atrial fibrillation, deep vein thrombosis, CHF, chronic obstructive pulmonary disease or asthma. The impact of these diseases is more aggressive on dialysis patients than others. One study found that the GFR was lower after COVID-19 exposure (8). The mortality rate in COVID-19 dialysis patients was higher than in non-dialysis patients (9).

There are 20 hemodialysis units in Palestine which include 12 units in the West Bank of which 11 are located at ministry of health hospitals that include 255 hemodialysis machines, while the remaining unit belongs to An-Najah National University private hospital which includes 45 hemodialysis machines (1). In 2021, there were 1,567 patients that received regular hemodialysis services in the West Bank (1). The hemodialysis unit at Palestine Medical Complex (PMC) provides regular hemodialysis to patients in the Ramallah area. Hemodialysis patients are in regular contact with other patients and healthcare provider which makes them at a higher risk of contracting infections. This study aims to examine the effects of COVID-19 on the livelihood and dialysis frequency in PMC hemodialysis patients in Palestine, and to examine related risk factors.

## 2. Materials and methods

### 2.1. Study design and population

In this study, a cross-sectional design was implemented. Data was collected retrospectively from the dialysis department in PMC in Ramallah during April 2022, the Avicenna medical records program was used to retrieve patients’ data. The study was approved by the research ethics committee at the college of Pharmacy at Birzeit University, approval number BZU-PNH-2135. The informed consent requirement was waived as data was collected from electronic records. The approval of the Palestinian Ministry of Health and PMC was also obtained prior to data collection. Patients who were on dialysis and who have been infected with COVID-19 between January 2020 and April 2022 were included in the study. The effect of COVID-19 on dialysis patients was examined and whether there were any exacerbations of the disease.

The collected data included patient demographics like gender, age, blood group, clinical features such as any comorbidities, COVID-19 symptoms, lab tests such as blood urea nitrogen (BUN), SCr, calcium, potassium and the frequency of dialysis, that is for how long the dialysis was applied and how many times it was repeated per week. All data was obtained from electronic medical records in the hospital.

The study included all hemodialysis patients at PMC who have been infected with COVID-19 since the beginning of the spread of the virus in Palestine till April 2022. 103 patients were included in the study, from different age groups, that were diagnosed with end-stage renal disease requiring dialysis, the patients were receiving chronic maintenance hemodialysis and were diagnosed with acute COVID-19 infection.

### 2.2. Statistical analysis

Data was introduced, filtered and separated in excel worksheets, then transferred and analyzed using IBM Statistical Package for the Social Sciences (SPSS) program version 22. The descriptive analysis was performed, mean and standard deviation were calculated for continuous data, while frequencies and percentages were computed for categorical data. Chi-square test and fisher exact test were applied to assess if there were any significant associations among variables. Chi-square test, with 95% confidence interval, was performed to assess the presence and absence of association between the frequency of dialysis and many other variables such as age, blood group, comorbidities and electrolyte values. Then, binary logistic regression, enter method, was applied for all variables with a value of *p* <0.05 to investigate the confounders.

## 3. Results

### 3.1. Patients demographic and clinical characteristics

Patients receiving hemodialysis services at PMC kidney dialysis department that were infected with COVID-19 between 2020–2022 were included in this study (*n* = 103), the most common age group within the study sample was the group of 60 and above years old, which represented 42.7% of the sample, with the mean age ± SD (53.378 ± 15.71). 60.2% of patients in the study were males and 39.8% were females. The O blood group accounted for the highest percentage of all blood groups (41.7%). It is important to mention that 68% of patients remained alive at the time of data collection while 32% died, the demographic data of the patients is shown in Table 1.

**Table 1:** Demographic and clinical characteristics of the study sample with and without change in their dialysis frequency before and after COVID-19 infection.

		No change in dialysis^+^ *n* (%)	Change *n* (%)
Gender	Male	15 (46.9)	47 (66.2)
	Female	17 (53.1)	24 (33.8)
Alive	Yes	32 (100)	38 (53.5)
	No	0	33 (46.5)
Blood groups	A	9 (28.1)	28 (39.4)
	B	2 (6.3)	5 (7.0)
	AB	4 (12.5)	12 (16.9)
	O	17 (53.1)	26 (36.6)
Age (years)	<40	8 (25)	17 (23.9)
	40–59	7 (21.9)	27 (38)
	≥ 60	17 (53.1)	27 (38)
BUN mg/dL	Low	1 (3.1)	0
	Normal	0	13 (18.3)
	High	31 (96.9)	58 (81.7)
Calcium (Ca) mg/ dL	Hypocalcemia	16 (50)	52 (73.2)
	Normal	16 (50)	19 (26.8)
	Hypercalcemia	0	0
Potassium (K) mmol/L	Hypokalemia	1 (3.1)	4 (5.6)
	Normal	16 (50)	41 (57.7)
	Hyperkalemia^†^	15 (46.9)	26 (36.6)
SCr mg/dL	Normal	0	5 (7)
	High	32 (100)	66 (93)
DM	Yes	10 (31.3)	30 (42.3)
	No	22 (68.7)	41 (57.7)
Hypertension	Yes	11 (34.4)	7 (9.9)
	No	21 (65.6)	64 (90.1)
Kidney transplant	Yes	1 (3.12)	5 (7)
	No	31 (96.9)	66 (93)
Angina Pectoris (AP)	Yes	4 (12.5)	4 (5.6)
	No	28 (87.5)	67 (94.4)
IHD	Yes	7 (21.9)	10 (14.1)
	No	25 (78.1)	61 (85.9)
CHF	Yes	5 (15.6)	13 (18.3)
	No	27 (84.4)	58 (81.7)
Benign neoplasm	Yes	9 (28.1)	9 (12.7)
	No	23 (71.9)	62 (87.3)

+These patients were already undergoing the maximum number of hemodialysis which is 3 per week according to the PMC kidney unit protocol, after their exposure to COVID-19 the frequency of dialysis did not change and there was no further information that can rule out whether the dialysis did not change because it is at its maximum number or the patients’ situation did not change. ^†^Hyperkalemia: blood potassium levels > 5.2 mmol/L.

**Figure 1:**
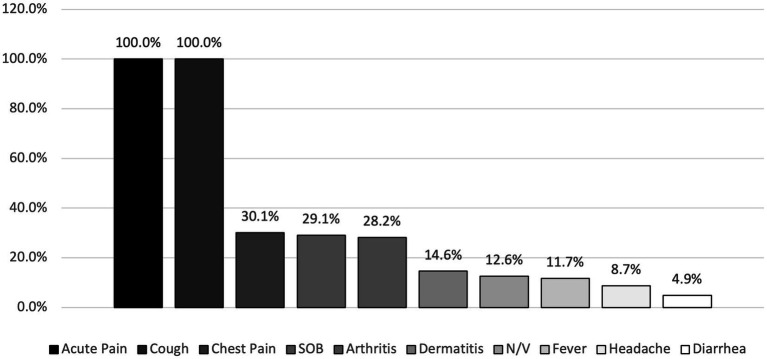
Percentages of COVID-19 symptoms among hemodialysis patients.

As shown in Figure 1, all hemodialysis patients suffered from cough and acute pain during their COVID-19 infection period, with a high percentage of SOB and chest pain, accounting for 30.1 and 29.1% respectively, reported symptoms also included arthritis (28.2%), dermatitis (14.6%), N/V (12.6%), fever (11.7%), headache (8.7%), and diarrhea (4.9%).

**Figure 2:**
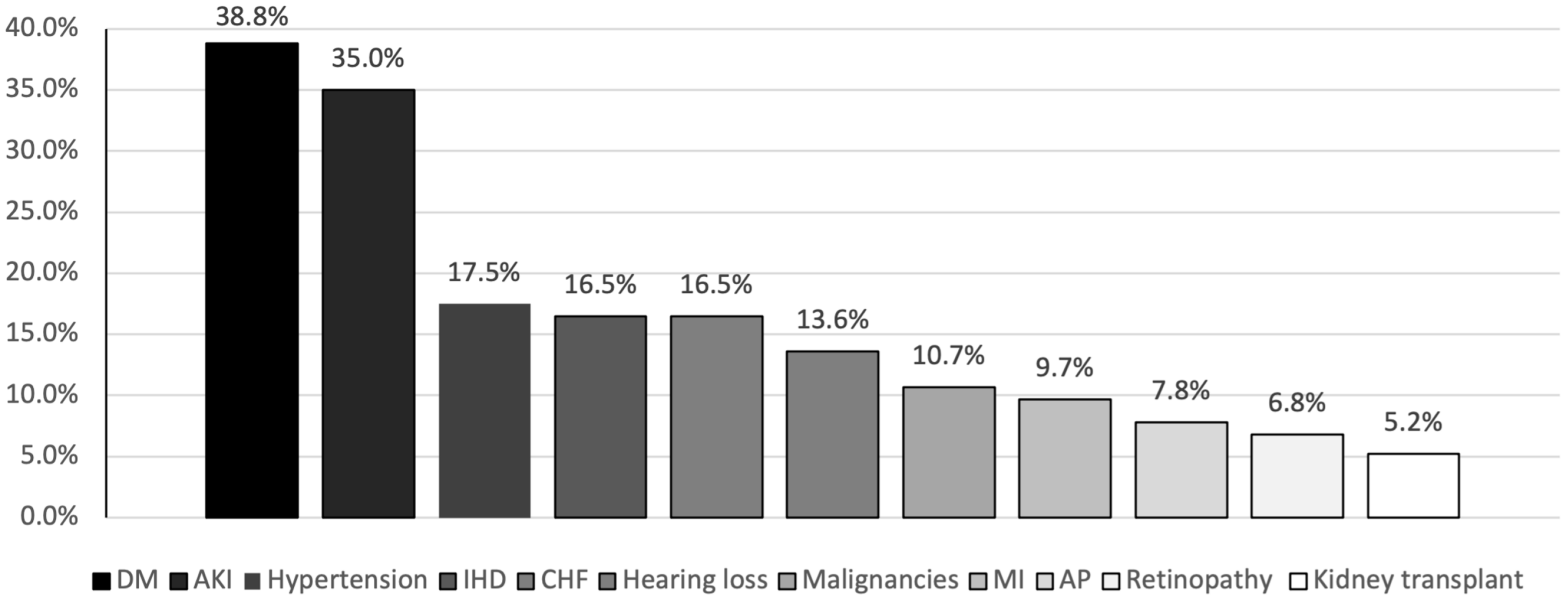
Percentages of comorbidities that COVID-19 infected hemodialysis patients suffered from.

The most common comorbidity in the study sample was DM which was found in 38.8% of patients, followed by hypertension (17.50%), CHF and ischemic heart disease (IHD) both accounted for a percentage of 16.5% (Figure 2).

### 3.2. Patients’ outcomes

**Figure 3:**
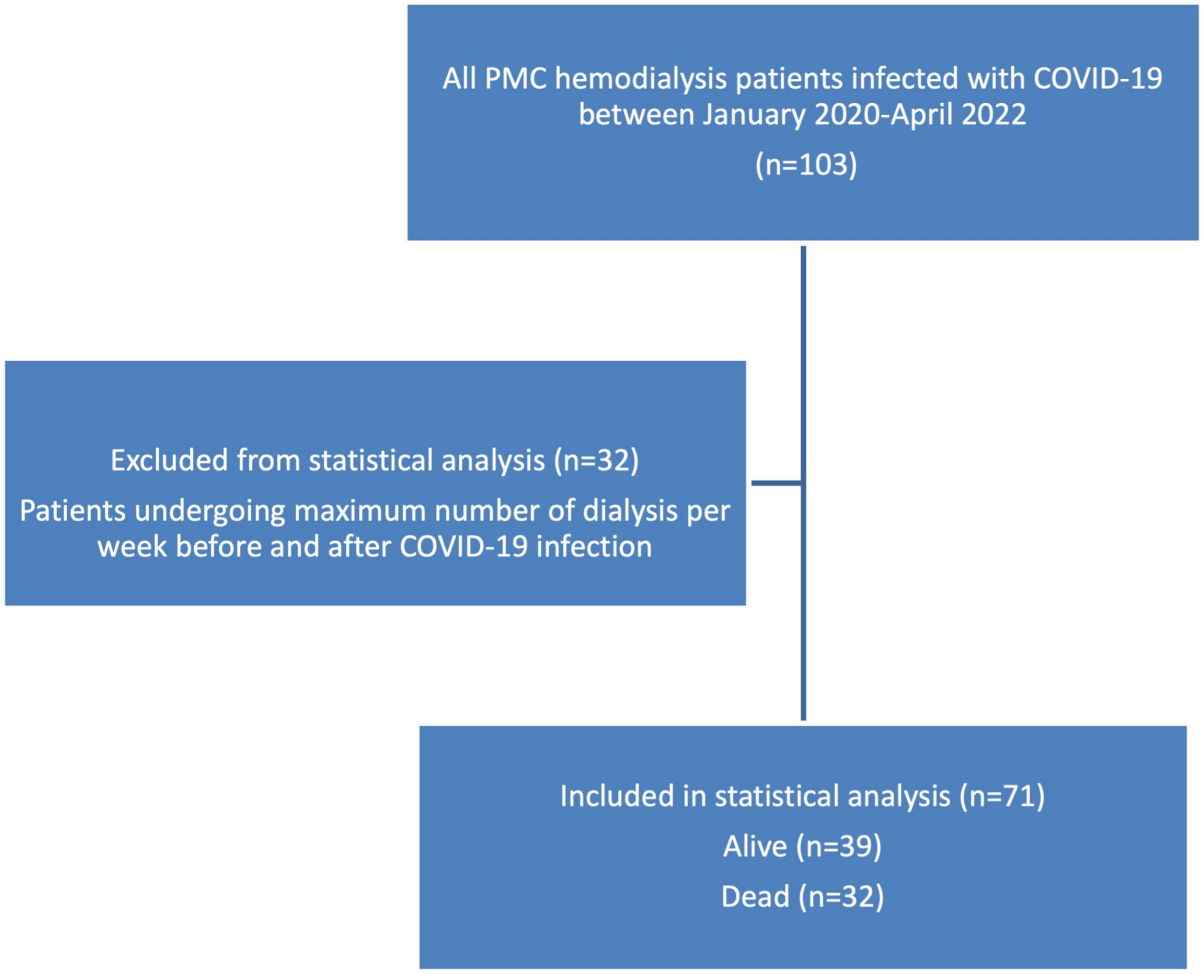
Scheme of inclusion criteria in statistical analysis.

An important objective of this study is to examine the impact of COVID-19 on the frequency of hemodialysis. Within the study sample, 32 patients have already reached the maximum number of dialysis per week before their COVID infection, which is 3 times per week as per the PMC kidney unit protocol, and their dialysis frequency remained the same after their COVID-19 recovery, there was not enough information in the medical records to conclude whether their need for dialysis was not increased post COVID, or their frequency of dialysis was not changed because it has reached its maximum already before the COVID-19 infection, these patients were sorted out of the further analysis since there was not enough information in their medical records for them to be included in the statistical analysis (Figure 3). As for the rest of the patients, 32 died, and the remaining 39 patients all had an increase in their dialysis frequency, the characteristics and clinical conditions of these 2 subgroups are found in Table 2.

**Table 2:** The characteristics and outcomes on hemodialysis for patients that remained alive and had changes in the frequency of dialysis or died after their COVID-19 infection.

		Alive *n* (%)	Dead *n* (%)	Unadjusted *p*-value	Adjusted *p*-value	Adjusted OR (CI 95%)
Gender	Male	25 (53.2)	22 (46.8)	0.680	Not entered	
	Female	14 (58.3)	10 (41.7)			
Blood groups	A	22 (78.6)	6 (21.4)	0.006*	Ref.	
	B	3 (60)	2 (40)		0.824	1.44 (0.06–34.8)
	AB	5 (41.7)	7 (58.3)		0.193	3.69 (0.52–26.4)
	O	9 (34.6)	17 (65.4)		0.011*	10.5 (1.72–64.14)
Age (years)	<40	12 (70.6)	5 (29.4)	0.236	Not entered	
	40–59	15 (55.6)	12 (44.4)			
	≥60	12 (44.4)	15 (55.6)			
BUN mg/dL	Normal	1 (7.7)	12 (92.3)	<0.001*	Ref.	0.0 (0.0-…)
	High level	38 (65.5)	20 (34.5)		0.999	
Calcium (Ca) mg/ dL	Normal	15 (78.9)	4 (21.1)	0.014*	Ref.	2.29
	Hypocalcemia	24 (46.2)	28 (53.8)		0.307	(0.468–11.213)
Potassium (K) mmol/L	Hypokalemia	1 (25)	3 (75)	0.498	Not entered	
	Normal	24 (58.5)	17 (41.5)			
	Hyperkalemia	14 (53.8)	12 (46.2)			
SCr mg/dL	Normal	1 (20)	4 (80)	0.037*	Ref.	91,413,501 (0.0-…)
	High level	38 (57.6)	28 (42.4)		0.999	
DM	Yes	12 (40)	18 (60)	0.031*	Ref.	0.121 (0.022–0.67)
	No	27 (65.9)	14 (34.1)		0.016*	
Hypertension	Yes	7 (100)	0	0.014*	Ref.	3,333,775,125 (0.0-…)
	No	32 (50)	32 (50)		0.999	
Kidney transplant	Yes	2 (40)	3 (60)	0.652	Not entered	
	No	37 (56.1)	29 (43.9)			
IHD	Yes	5 (50)	5 (50)	0.764	Not entered	
	No	34 (55.7)	27 (44.3)			
CHF	Yes	5 (38.5)	8 (61.5)	0.187	Not entered	
	No	34 (58.6)	24 (41.4)			
Benign neoplasm	Yes	4 (44.4)	5 (55.6)	0.722	Not entered	
	No	35 (65.5)	27 (43.5)			
Visual disturbances	Yes	5 (45.5)	6 (54.5)	0.527	Not entered	
	No	34 (56.7)	26 (43.3)			
MI	Yes	1 (12.5)	7 (87.5)	0.019*	Ref.	0.638 (0.03–13.553)
	No	38 (60.3)	25 (39.7)		0.773	
Arthritis	Yes	13 (59.1)	9 (40.9)	0.637	Not entered	
	No	26 (53.1)	23 (46.9)			

*Results were considered significant when *p* < 0.05. Increasing dialysis frequency was set as reference for binary logistic regression test.

Looking at the electrolyte levels analysis after COVID, chi-square test results revealed that the BUN, SCr and calcium had significant associations with the frequency of dialysis, (*p <* 0.001) and (*p* = 0.037) for BUN and SCr respectively, hence COVID-19 contributes to increasing the BUN and SCr levels leading to more dialysis treatments (Table 2). BUN and SCr were also high in the patients that were excluded from statistical analysis and whose dialysis frequency did not change as it was at its maximum before COVID (Table 1). For the 46.2% of patients that had a decrease in calcium level there was an increase in dialysis treatments per week (*p* = 0.014), as for the potassium levels, there was no association with the frequency of dialysis (*p* = 0.498) (Table 2).

Regarding comorbidities, there was a significant association between the frequency of dialysis and hypertension (*p* = 0.014), for all patients that had hypertension within the study sample and got infected with COVID-19 the frequency of dialysis was increased. MI also had a significant association with the frequency of dialysis (*p* = 0.019) according to chi-square test results. For the 8 patients that had MI 7 have died and one had an increase in dialysis frequency. There was no significant association with other comorbidities such as IHD and CHF (Table 2).

Multivariate analysis revealed a significant association between the patient’s blood group and being diagnosed with DM and the dialysis frequency. Patients with the O blood group were ten folds more likely to die compared to patients with the A blood group (OR = 10.5, CI 95% = 1.7–64.1). While non-diabetic patients were significantly less likely to die compared with diabetic patients (OR = 0.121, CI 95% = 0.02–0.67). No significant associations were found between the dialysis frequency and the other tested variables.

## 4. Discussion

This study examined the clinical course and outcome of COVID-19 infection in hemodialysis patients in PMC. It included all patients from PMC kidney dialysis unit infected with COVID. Data was collected for 103 patients undergoing hemodialysis with laboratory-confirmed COVID-19 diagnosis from the beginning of the pandemic until the end date for data collection, between January 2020–April 2022.

The findings of this study showed that COVID-19 adversely impacted hemodialysis patients, the study population was divided into 3 groups, a group that died after COVID-19 accounting for 31% of the patients, a group that survived after the infection but their frequency of dialysis has increased (36.4%), and the third group (33.6%) was already at the maximum number of weekly dialysis before COVID-19, and remained on the same number of dialysis post COVID-19 infection, this group was excluded from the statistical analysis, as it could not be ruled out whether the number of dialysis remained the same because it was at its maximum per the hospital protocol, or because there was no need for the increase.

In our study 32 patients died after their infection with COVID-19 with a percentage of 31%, a study in HD units in Madrid, Spain reported that dialysis constituted the highest risk factor of death for COVID-19 patient during the first month of diagnosis, similarly in our study the patients died immediately after they got the infection (10). Other studies also found that the morbidity and mortality following COVID-19 infection was a lot higher in hemodialysis patients than in the general population (11–14).

Symptoms of COVID-19 in this study sample included acute pain in different body parts in addition to cough in all patients, other symptoms occurred at varying percentages such as SOB, chest pain, headache, arthritis, dermatitis, fever and diarrhea (Figure 1). Even though these are the general symptoms that also exist in non-dialysis patients, hemodialysis patients have worse symptoms of COVID-19 because they tend to be elderly and to have multiple comorbid conditions, like hypertension, DM, coronary artery disease, CKD, heart failure and suppressed immune systems (15). In our study, the recorded symptoms were mild to moderate and drugs have been used to alleviate these symptoms including antibiotics like azithromycin, clindamycin, ceftazidime, cefuroxime, etc., as well as pain management, antipyretic and anti-inflammatory medications including paracetamol, aspirin, hydrocortisone, and various vitamins and mineral supplements.

For the patients included in statistical analysis, the A blood group was the most common and they were significantly affected with an increase in the frequency of dialysis after having the COVID-19 infection. Previous studies that were performed in many countries such as China and Canada have shown a significant association between the A blood group and the complications after COVID-19 infection (16). Another comprehensive review also found that the A blood type was associated with more COVID infection susceptibility while the O blood type was more protective (17). The proposed mechanism behind this is that the patients with group A blood type have higher levels of ACE 1 and ACE 2, which increases the affinity of SARS-COV-2 to theses receptors resulting in more severe infection, while the O group produces anti-A antibodies that bind with A-like antigen, which is created from SARS-COV-2 virus envelope, resulting in the prevention of infection (16). Another suggested mechanism is the inhibition of the virus adhesion to the host cell via two ways either by anti-A antibody binding to the SARS-COV-2 S protein, and blocking the interaction between this protein and ACE 2 receptors to prevent the virus from lung entry, or via SARS-COV-2 glycan antigen, which is similar to antigen A but does not exist in the O blood group, so it is proposed that the patients with the A blood group have a greater risk of COVID-19 severity as they lack anti-A antibodies (16). While the previously mentioned studies found that patients with the O group had the least severity and mortality, in our study the O blood group was associated with a higher percentage of fatality (Table 2). A study on the effects of COVID-19 on recovered patients in Palestine found no association between the blood groups and post COVID outcomes (18). The issue of blood group and severity of COVID-19 complications has not been resolved yet with different studies showing controversial results (16).

Approximately, more than half of the infected patients were males (60%), this is probably because males in Palestine tend to be more socially active than females, and according to many previous studies that had been done in many different countries, it was reported that women were more responsible in dealing with the COVID-19 pandemic than men, who had an irresponsible attitude that manifested by their lower rate of hand washing and wearing face masks (19). Also, it was shown that males had a higher risk of death, as two thirds of dead patients were males (19), but in our study this was not statistically significant (*p* = 0.687) (Table 2).

As for the age, there was no statistically significant difference between the age groups (*p* = 0.236). Many comorbidities were recorded with a significant association with dialysis frequency changes, this outcome can be justified by the age of the patients included in the study as most of them were elderly and suffered from chronic diseases. According to a nationwide analysis in China, COVID-19 patients with underlying comorbidities had more severe COVID-19 infection and poorer clinical outcomes compared to patients without comorbidities (20).

The most common comorbidity recorded in our study was DM, which significantly affected the outcome on hemodialysis patients increasing the frequency of dialysis or mortality (*p* = 0.031), DM is a risk factor for kidney failure as high levels of sugar in the blood damage the filtering units in kidney, which can lead to a significant damage including end stage kidney failure that requires dialysis (8).

In August 2020, WHO announced that there was an association between cardiovascular disease and COVID-19, as there was an increased risk of both arterial and venous thrombotic complications, and after 7 days of COVID-19 diagnosis the risk of MI was doubled (21). A study in the city of Mus in Turkey recommended that patients on dialysis should take prophylactic anticoagulant and antiplatelet agents while they are infected with COVID-19 (22). Despite the risk of bleeding, the risk of thrombosis is still considered higher compared to the general population. A study found that COVID-19 patients had a higher risk of clotting in extracorporeal circuits (23). According to our study, multivariate analysis showed no significant association between acute MI and COVID-19 outcomes in dialysis patients, this could be attributed to the small number of MI patients within the study sample. Most of the patients in our study were on aspirin or other anticoagulant therapy like enoxaparin, this is important as hemodialysis patients require special attention to prevent the risk of thromboembolic events including MI (22, 24).

Another cardiovascular comorbidity that has a significant correlation with dialysis and COVID-19 outcomes is pre-existing hypertension. The findings of many observational studies in China demonstrated that the majority of the COVID-19 patients with hypertension were at a higher risk of developing severe outcomes of dialysis deterioration, one of them, is the increase in dialysis frequency (25). All of the hypertensive patients in our study had an increase in the number of dialysis sessions after COVID-19 infection, however, none of the hypertension patients died. There are two suggested explanations for this either via the renin angiotensin system, or by causing endothelial dysfunction due to the production of high levels of pro-inflammatory agents such as angiotensin II, cytokines, interleukin-6 and tumor necrosis factor-α causing an imbalance between relaxing and constrictor factors, moreover, hypertension is one of the main risk factors for kidney dysfunction, if the blood pressure is not controlled, it can lead to acute kidney injury (AKI) in COVID patients (25, 26). Nevertheless, more studies are required to confirm the association between hypertension and increased risk of complications in dialysis patients affected by COVID-19. AKI was defined, based on KDIGO classification, as a change in either SCr, by 0.3 mg/dL over two days or an increase in SCr 1.5 times more than the baseline within the last 7 days, or urine output less than 0.5 mL/kg/h. for 6 h. In addition, being elderly with hypertension and DM are considered as risk factors for AKI. COVID-19 targets ACE receptors which can be associated with adverse clinical outcomes in COVID patients including AKI (27). Various studies indicate a significant association between kidney abnormalities and COVID-19 complications (27–29).

Our study included six patients who had a kidney transplant, and after COVID-19 infection 3 of them died and 3 continued to live with kidney failure, according to a multi-center study done in France, a high mortality rate was reported among the recipients of kidney transplant who got coronavirus infection (30).

Both SCr and BUN are indicators for kidney function. SCr normal level is between 0.59 to 1.35 mg/dL and for BUN, it is between 6–24 mg/dL. According to our study, more than half of the patients who had a high BUN and raised level of SCr had an increase in their dialysis frequency (Table 2).

Our results show that lower serum calcium in patients with COVID-19 increased dialysis frequency (Table 2), while the mechanism behind this is still not fully resolved, a study demonstrated that SARS-COV specific gene (E) encodes a small protein with a permeable calcium channel that causes more calcium entry into the cells (31).

In our study, there were five patients with hypokalemia, three of them died after COVID-19 infection, the number of patients is too small and the association was not statistically significant (*p* = 0.498), another study found that hypokalemia was common among COVID-19 patients and it was accompanied with hypocalcemia (32).

This study had some limitations. It was a retrospective study because the restrictions imposed during the COVID pandemic limited researchers’ access to hospitals, which was particularly problematic during the long periods of complete lockdown. In addition, the outcome data is relevant to similar hemodialysis centers that offer a maximum of 3 dialysis treatments per week. Furthermore, some data was missing from patients’ medical records and could not be retrieved at the time of data collection including eGFR, dialysis time, and urea reduction ratio. The missing data could have been a result of the high workload combined with the shortage of healthcare staff during the COVID pandemic.

In conclusion, among all PMC hemodialysis patients infected with COVID-19, there was a higher fatality rate than observed in the general population. Moreover, there was a significant correlation between comorbidities such as DM and hypertension and increased dialysis frequency, hence the comorbidities should be kept under control as much as possible in hemodialysis patients. As the precautions and restrictions for COVID-19 are decreasing worldwide and in Palestine in particular, care should be given to highly vulnerable populations including hemodialysis patients. In addition, more studies need to be conducted on complications of infections and other comorbidities in hemodialysis patients, and regular screening tests as well as other preventive measures should be practiced to protect hemodialysis patients.

## Data availability statement

The raw data supporting the conclusions of this article will be made available by the corresponding author upon request without undue reservation.

## Ethics statement

The studies involving humans were approved by Research ethics committee at the college of Pharmacy at Birzeit University. The studies were conducted in accordance with the local legislation and institutional requirements. Written informed consent for participation was not required from the participants or the participants’ legal guardians/next of kin in accordance with the national legislation and institutional requirements.

## Author contributions

LA: Conceptualization, Project administration, Supervision, Validation, Writing – original draft, Writing – review & editing. TA-S: Conceptualization, Data curation, Formal analysis, Methodology, Writing – original draft. SS: Conceptualization, Data curation, Formal analysis, Methodology, Writing – original draft. NA-S: Formal analysis, Writing – review & editing.
